# Measurement error in bioimpedance spectroscopy: The role of electrode type and analyzer

**DOI:** 10.2478/joeb-2026-0012

**Published:** 2026-08-25

**Authors:** Carina M. Velasquez, Ainsley E. Way, Christian Rodriguez, Ethan G. Tinoco, Christine M. Florez, Madelin R. Siedler, Todd J. Freeborn, Grant M. Tinsley

**Affiliations:** Energy Balance & Body Composition Laboratory, Dept. of Kinesiology and Sport Management, Texas Tech University, Lubbock, TX, USA; Pennington Biomedical Research Center, LSU System, Baton Rouge, LA, USA; Department of Exercise and Health Science, College of Saint Benedict and Saint John's University, Saint Joseph, MN, USA; Department of Electrical and Computer Engineering, The University of Alabama, Tuscaloosa, AL, USA

**Keywords:** Bioimpedance, body composition, electrical impedance, electrodes

## Abstract

Bioimpedance spectroscopy (BIS) is a non-invasive method used to assess body composition in a variety of settings. Although BIS has been reported as an accurate measure of body composition, the magnitude of within-day technical error associated with different BIS devices and electrode types remains unknown. Therefore, the purpose of this study was to assess the technical error of measurement (TEM) between single-tab and double-tab electrodes across two BIS devices for resistance, reactance, phase angle, total body water, fat-free mass, and fat mass. One hundred and four adults reported to the laboratory for a single visit, undergoing twelve different assessments utilizing single- and double-tab electrodes on two BIS analyzers of the same model. The within-day technical error was assessed as the absolute and relative TEM for each of the variables. The results indicated that intra-analyzer assessments displayed the lowest amount of error for all the variables (relative TEM = 0.05 – 0.28%) while the largest TEMs were observed for comparisons between single-tab and double-tab assessments (relative TEM = 1.04 – 3.68%), except for reactance. For reactance alone, the TEMs were greatest for the inter-analyzer comparison with double-tab electrodes (absolute TEM = 1.07 Ω; relative TEM = 1.68%). While all assessments displayed relatively low technical error, the present results reinforce the importance of consistently using the same analyzer and electrode type to minimize errors when assessing body composition using BIS, particularly when seeking to identify subtle changes in BIS output.

## Introduction

Bioimpedance spectroscopy (BIS) is a non-invasive and quick method used to estimate body composition. BIS has grown in popularity within the clinical and research setting, as it allows for easy assessment of cross-sectional and longitudinal evaluations of body composition in diseased populations, resistance-trained individuals, and nutritional intervention studies (1,2,3,4). Moreover, BIS has been used to estimate different body water compartments when whole-body dilution techniques (i.e., deuterium oxide [D_2_0 or ^2^H]) are not feasible (5, 6). The use of BIS allows researchers to estimate total body water (TBW), which can be further used to derive body composition metrics like fat-free mass (FFM) and fat mass (FM) (3, 5, 7).

**Figure 1.**
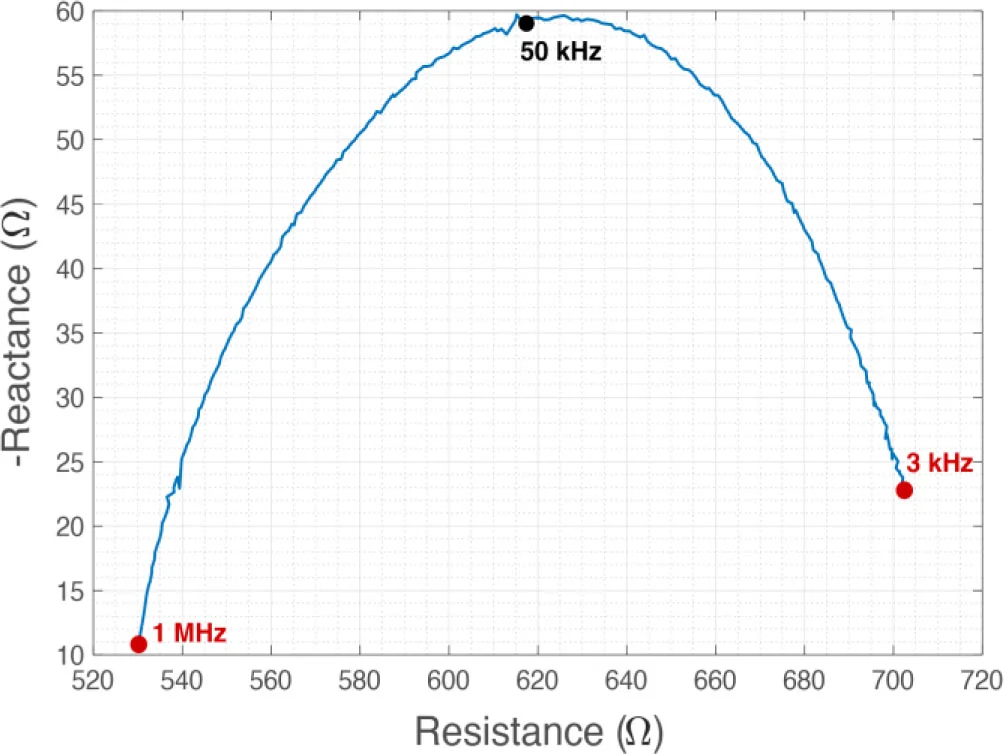
Sample hand-to-foot multi-frequency bioimpedance collected from a healthy adult. Red markers highlight the low (3 kHz) and high (1 MHz) frequency datapoints. The black marker highlights 50 kHz data, which is the specific frequency analyzed in this study.

While both body composition and body fluids can be estimated by BIS, the underlying properties of the body directly measured by BIS are the ‘raw’ bioimpedance variables. The term bioimpedance refers to the electrical impedance (Z = R + jXc) of a biological tissue which is a complex value with resistance (R) and reactance (Xc), where j denotes the imaginary number (j = √−1). The resistance (or real component of the bioimpedance) reflects the opposition to the flow of an electrical current through the tissue of the body, while the reactance (or imaginary component of the impedance) is attributed to the tissue interface and cell membrane integrity, indicative of a delayed response in the conduction of the electrical current due to the capacitive properties of the cell membrane (2,3,4, 8). From the aforementioned variables, phase angle (*φ*) can be determined as *φ* = tan^−1^(Xc/R)⋅(180/π), representing the angular relationship between R and Xc within the R-Xc vector, and is often used as an indicator of cellular membrane integrity (9). Although BIS employs many frequencies, 50 kHz is often a focus when evaluating raw bioelectrical variables. To illustrate this, the 3 kHz to 1 MHz hand-to-foot bioimpedance collected from a healthy adult is presented in Fig. 1 with the 50 kHz value denoted with a black circle.

Previous studies (10,11,12), have demonstrated the frequent use of *φ* for comparing individual values to reference data, thereby providing a prognostic understanding in clinical populations. Given the growing application of BIS, methodological factors influencing the reliability of its output variables warrant careful consideration. Moon et al. (5) examined the between-day reproducibility of R and TBW estimates utilizing single- and double-tab electrodes, reporting that the double-tab electrodes (i.e., a fixed distance electrode configuration of 5 cm) reduced error in TBW as compared to standard electrode sites using single-tab electrodes. Moreover, in a previous study conducted by Donahue et al. (13), the authors evaluated the consistency of BIS measurements utilizing single-tab and double-tab electrodes to assess patients with lymphedema; the authors noted that consistency of BIS measurement were not dependent on the electrode type and no significant differences between electrode types were observed. While Moon et al. (5) and Donahue et al. (13) collectively provided important insight into the between-day reliability and consistency of BIS measures when employing differing electrode types, the magnitude of within-day technical error associated with different BIS devices and electrode types in a healthy population remains unclear. Therefore, the purpose of this study was to assess the technical error of measurement between single-tab and double-tab electrodes across two BIS devices for raw bioimpedance metrics and estimated body fluid and body composition outputs.

## Materials and methods

### Participants

Generally healthy adults were recruited to participate in this study, and prospective participants were excluded if they were missing any limbs or parts of limbs, had undergone a body-altering surgery (i.e., breast augmentation, liposuction, etc.), had any implanted metal due to a previous medical procedure, had a pacemaker or electrical implant, or were currently pregnant or trying to become pregnant. One hundred and four adults (twelve Asian, six Black/African American, twenty-four Hispanic/Latino, sixty-two White), including fifty-six females (mean ± SD age = 22.8 ± 6.7 years; body mass 66.3 ± 13.4 kg; height = 164.03 ± 5.8 cm; BMI = 24.7 ± 4.9 kg/m^2^) and forty-eight males (mean ± SD age = 24.4 ± 7.8 years; body mass 79 ± 14 kg; height = 176.7 ± 6.9 cm; BMI = 25.3 ± 4.1 kg/m^2^), reported to the laboratory for one visit. Participants reported to the laboratory after following an overnight (≥8 hours) abstention from eating, drinking, exercising, and ingestion of any caffeine or alcohol. Prior to the start of the visit, urine specific gravity (USG) was assessed with a digital refractometer (PA201X-093, Misco, Solon, OH, USA) to provide an indication of hydration status (mean ± SD 1.02 ± 0.006).

### Informed consent

All participants provided written informed consent prior to participation.

### Ethical approval

This study was approved by the Texas Tech Institutional Review Board (Protocol #2022-610) and adhered to the ethical principles of the Declaration of Helsinki.

### ImpediMed SFB7

Bioimpedance spectroscopy was assessed using two ImpediMed SFB7 (ImpediMed Inc. Pinkenba, QLD, Australia) instruments. These devices use a tetra-polar configuration to measure the multi-frequency bioimpedance of a participant or tissue with discrete frequencies between 3 kHz and 1 MHz. In the tetrapolar configuration, two electrodes drive a low-level sinusoidal electrical current (I) into the tissue and two electrodes sense the excited voltage (V). Using these values, the tissue impedance (Z = V / I) is calculated for each discrete frequency. The multi-frequency bioimpedance is used along with the details of the participant’s sex, age, height, and weight to estimate their body composition. In the present study, all collected measurements were exported from the SFB7 devices using the ImpediMed’s BioImp (v 2.0.1) software.

### Ag/AgCl Electrodes

Single and double-tab Ag/AgCl electrodes from ImpediMed were used to interface the SFB7 devices to participants to measure their electrical impedance. The single-tab electrodes contained a gel surface area of 575 mm^2^ (i.e., 25 mm length and 23 mm width). The double-tab electrodes had reported dimensions of 75 mm length and 23 mm width, with gel dimensions approximately equal to the single-tab variant's 575 mm^2^ (i.e., 25 mm length and 23 mm width).

### BIS Assessments

**Figure 2.**
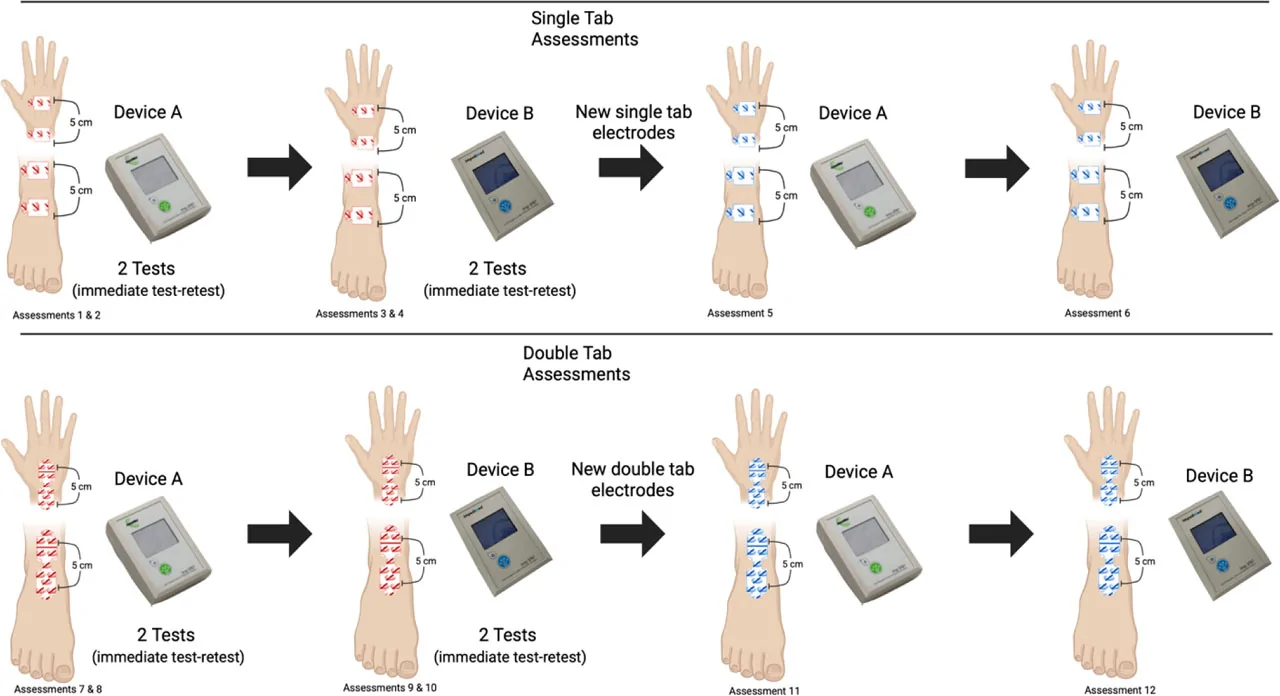
Study design of the twelve assessments utilizing single- and double-tab electrodes and the two different devices (i.e., device A and device B).

Prior to each day of testing, both BIS analyzers were tested using the manufacturer-provided calibration cell to ensure correct operation. Each participant underwent twelve different measurements to establish the technical error of the measure from different electrode types and analyzers, as displayed in Figure 2. Prior to beginning the first BIS assessment, participants lay in a supine position for approximately ~5 minutes to account for fluid stabilization (14). Prior to placing the electrodes, the electrode sites on the hands, wrist, foot, and ankle were cleaned with alcohol pads. The BIS electrodes were placed unilaterally on the right side of the body in accordance with the manufacturer guidelines. Before beginning the assessments, the participants’ limbs were spread apart to ensure there was no contact with other parts of the body. Participants remained motionless in the supine position during all of the assessments. For all assessments utilizing single-tab electrodes, a 5-cm measuring card was used to measure out the 5-cm distance between electrodes. The first assessment used two single-tab electrodes and device A (SFB7, ImpediMed Inc, Pinkenba, QLD, Australia; SN: U400-01E08119; green label), with the second assessment being an immediately repeated test using the same electrodes and analyzer (i.e., assessing intra-analyzer technical error for device A). The third assessment consisted of the same single-tab electrodes while utilizing device B (SFB7, ImpediMed Inc, Pinkenba, QLD, Australia; SN: SFB7-19D170012; blue label) for the measurement, followed immediately by an additional measurement with the same electrodes and analyzer to assess the intra-analyzer technical error for device B. For the fifth and sixth assessments, the electrodes utilized for assessments 1 – 4 were removed and replaced with a new set of single-tab electrodes, and a single measurement was completed using device A (assessment 5) and device B (assessment 6) to establish the error associated with using different sets of single-tab electrodes. For assessments 7 – 12, double-tab electrodes were utilized. The seventh assessment was performed with device A and consisted of double-tab electrodes being placed in the same position as the single-tab electrodes in accordance with the manufacturer’s guidelines. The eighth assessment was an immediate retest of device A to assess the intra-analyzer technical error of the measurement for the double-tab electrodes. For the ninth assessment, the same electrode set up from the prior double-tab assessment was applied to device B, with an immediate retest conducted afterwards (assessment 10; intra-analyzer for double-tab electrode on device B). For the eleventh and twelfth assessments, the double-tab electrodes used in the previous assessment were removed and replaced with a new set of double-tab electrodes, and single assessments were conducted using devices A and B, respectively. A summarized version of each assessment can be found in Table 1. Following each of the assessments, the bioelectrical output was processed using manufacturer-provided software (BioImp v. 2.0.1), and Cole-Cole plots were reviewed for basic quality assurance. For the present analysis, “measured”, rather than time delay (Td) corrected values, were used for raw bioimpedance metrics. The missing data rate was 0.56%, with 1,241 of 1,248 possible tests available. Reasons for missing data were occasional analyzer or data processing errors.

**Table 1.** A summary of the twelve comparisons utilizing single-tab (ST) and double-tab (DT) electrodes and the two different ImpediMed SFB7 analyzers (i.e., device A and device B). The colors in the table correspond to those displayed in Figures 3 – 8.

**Trials**	**Electrode Type(s)**	**Description**
**1 v 2**	**ST**	Test-retest with device A; intra-analyzer error
**1 v 3**	**ST**	Comparing devices A and B; inter-analyzer error
**3 v 4**	**ST**	Test-retest with device B; intra-analyzer error
**1 v 5**	**ST**	Comparing different sets of ST electrodes with device A; error from switching electrode sets
**3 v 6**	**ST**	Comparing different sets of ST electrodes with device B; error from switching electrode sets
**7 v 8**	**DT**	Test-retest with device A; intra-analyzer error
**7 v 9**	**DT**	Comparing devices A and B; inter-analyzer error
**9 v 10**	**DT**	Test-retest with device B; intra-analyzer error
**7 v 11**	**DT**	Comparing different sets of DT electrodes with device A; error from switching electrode sets
**9 v 12**	**DT**	Comparing different sets of DT electrodes with device B; error from switching electrode sets
**1 v 7**	**ST & DT**	Comparison of ST and DT electrodes for device A; error from switching electrode set/type
**3 v 9**	**ST & DT**	Comparison of ST and DT electrodes for device B; error from switching electrode set/type

### Statistical Analysis

The absolute technical error of measurement (TEM; i.e., precision error) and relative TEM (i.e., root-mean-square coefficient of variation) were used to establish the errors associated with each relevant comparison. The absolute TEM was calculated as $\sqrt{\frac{\Sigma \;D^{2}}{2n}}$ where D is the difference between the measurements and *n* is the sample size. The absolute TEM is represented in the original units of each variable. The relative TEM was calculated as the absolute TEM divided by the grand mean for the variable in question, multiplied by 100. The relative TEM is expressed as a percentage. The absolute and relative TEM were calculated for resistance, reactance, and phase angle at the 50 kHz frequency (R_50_, Xc_50_, and *φ*, respectively), total body water, fat-free mass, and fat mass. Data analysis was performed using R (v. 4.5.1).

## Results

### Reactance (Xc_50_)

**Figure 3.**
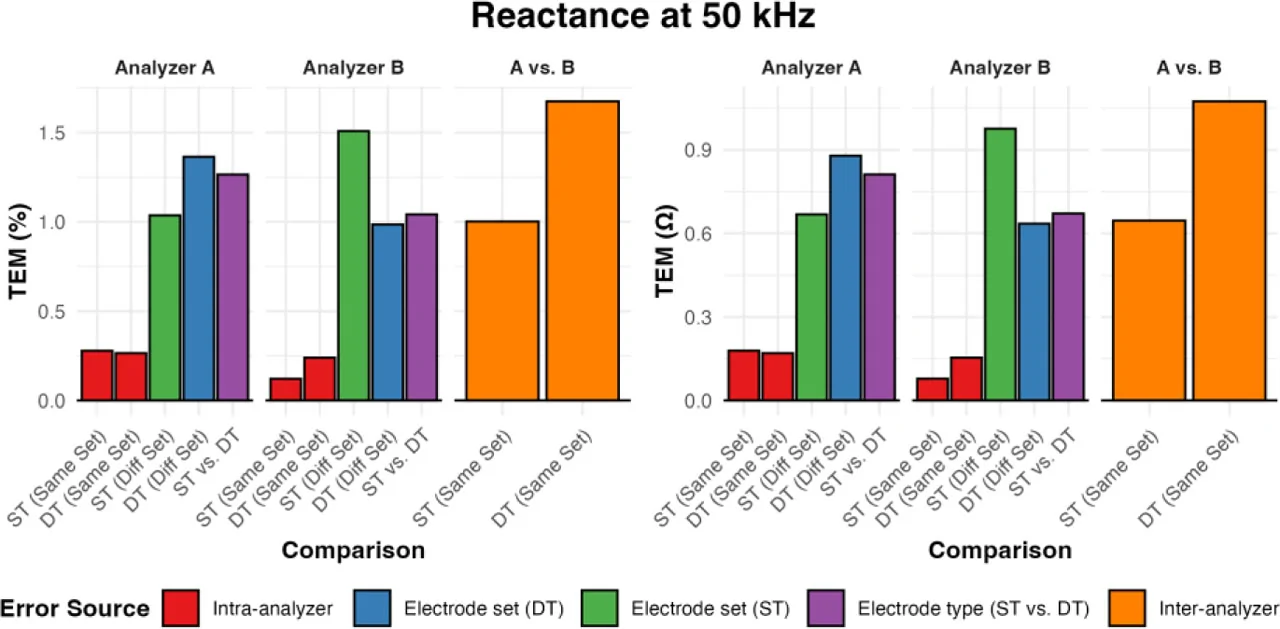
The technical error of the measurement (TEM) for reactance (Xc_50_) expressed in absolute (Ω) and relative (%) terms for each assessment, intra-analyzer in red (1 v. 2, 3 v. 4, 7 v. 8, 9 v. 10), different set of double-tab (DT) electrodes in blue (7 v. 11 and 9 v. 12), different set of single-tab (ST) electrodes in green (1 v. 5 and 3 v. 6), comparison of the different electrode types single vs double-tab electrodes in purple (1 v. 7 and 3 v. 9), and inter-analyzer in orange (7 v. 9 and 1 v. 3).

Immediate test-retest comparisons – indicating the intra-analyzer error – exhibited the smallest amount of absolute technical error ranging from 0.08 – 0.18 Ω and relative TEMs ranging from 0.12 – 0.28% (Table 2; Figure 3, red bars). When evaluated by electrode type, single-tab electrodes demonstrated absolute TEMs between 0.08 – 0.98 Ω and relative TEM values of 0.12 – 1.51% (Figure 3, red (ST), green, and orange (ST) bars). Double-tab electrodes had absolute TEMs of 0.15 – 1.07 Ω with relative TEM values of 0.24 – 1.68% (Figure 3, red (DT), blue, and orange (DT) bars). Across all assessments, the largest amount of error was observed in assessment 7 v. 9 (absolute TEM = 1.07 Ω; relative TEM = 1.68%), indicating relatively larger inter-analyzer technical errors with double-tab electrodes (Figure 3, orange bar (DT)).

**Table 2.** The absolute and relative technical error of the measurement for the raw bioelectrical impedance variables – Reactance (Xc_50_), Resistance (R_50_), Phase Angle (*φ*). See Figure 2 for assessment definitions.

**Assessment Comparison**	**Reactance (Xc_50_)**		**Resistance (R_50_)**		**Phase Angle (*φ*)**	
	Sample Mean ± SD 64.3 ± 8.6 Ω		Sample Mean ± SD 557.4 ± 100.6 Ω		Sample Mean ± SD 6.7 ± 1.0◦	
	**Abs. TEM (Ω)**	**Rel. TEM (%)**	**Abs. TEM (Ω)**	**Rel. TEM (%)**	**Abs. TEM (◦)**	**Rel TEM (%)**
1 v 2	0.18	0.28	0.30	0.05	0.02	0.27
1 v 3	0.65	1.00	1.37	0.25	0.05	0.8
3 v 4	0.08	0.12	0.26	0.05	0.01	0.11
1 v 5	0.67	1.04	6.59	1.19	0.06	0.95
3 v 6	0.98	1.51	6.37	1.15	0.09	1.31
7 v 8	0.17	0.27	0.31	0.06	0.02	0.24
7 v 9	1.07	1.68	1.19	0.21	0.09	1.38
9 v 10	0.15	0.24	0.62	0.11	0.02	0.24
7 v 11	0.89	1.36	6.37	1.13	0.09	1.33
9 v 12	0.63	0.98	6.34	1.13	0.08	1.13
1 v 7	0.81	1.27	7.80	1.40	0.11	1.64
3 v 9	0.67	1.04	7.92	1.42	0.10	1.45

### Resistance (R_50_)

**Figure 4.**
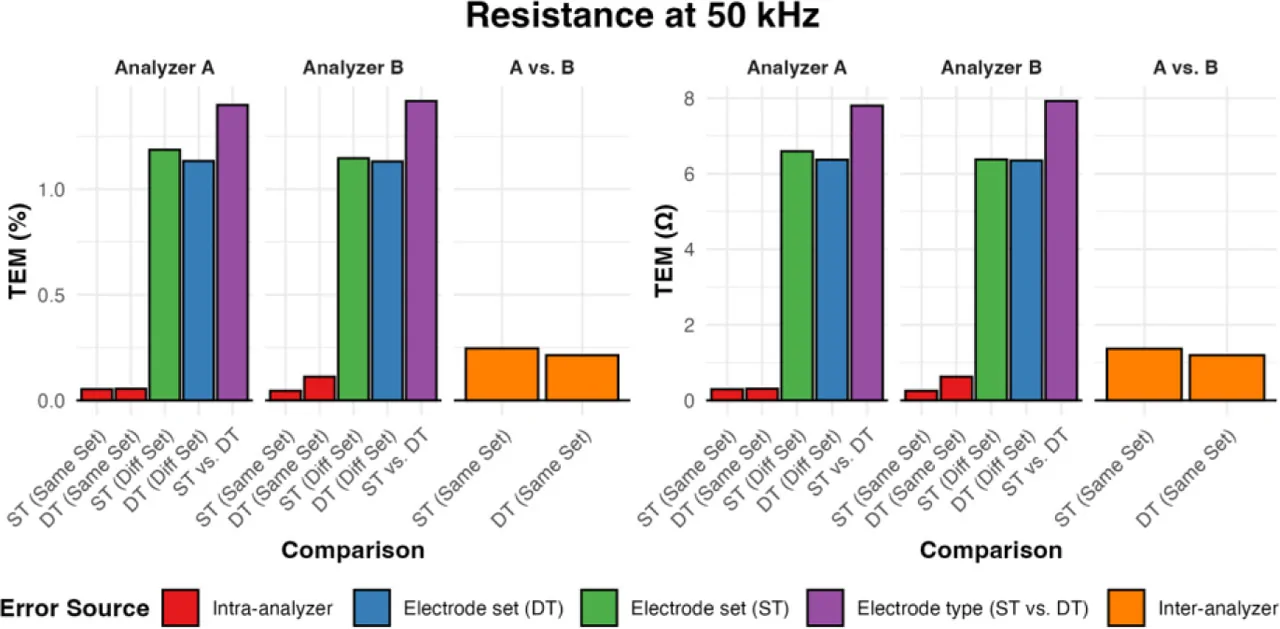
The technical error of the measurement (TEM) for resistance (R_50_) expressed in absolute (Ω) and relative (%) terms for each assessment, intra-analyzer in red (1 v. 2, 3 v. 4, 7 v. 8, 9 v. 10), different set of double-tab (DT) electrodes in blue (7 v. 11 and 9 v. 12), different set of single-tab (ST) electrodes in green (1 v. 5 and 3 v. 6), comparison of the different electrode types single vs double-tab electrodes in purple (1 v. 7 and 3 v. 9), and inter-analyzer in orange (7 v. 9 and 1 v. 3).

When evaluated by electrode type, the absolute TEM for single-tab electrodes ranged from 0.26 – 6.59 Ω with relative TEM values of 0.05 – 1.19% (Figure 4, red (ST), green, and orange (ST) bars). For the double-tab electrodes, the absolute TEM values were 0.31 – 6.37 Ω, with relative TEM values of 0.06 – 1.13% (Figure 4, red (DT), blue, and orange (DT) bars). When comparing different electrode types within the same analyzer (i.e., comparisons 1 v. 7 and 3 v. 9), larger errors were noted, with absolute TEMs of 7.80 – 7.92 Ω, and relative TEMs of 1.40 – 1.42% (Table 2; Figure 4, purple bars).

### Phase Angle (*φ*)

**Figure 5.**
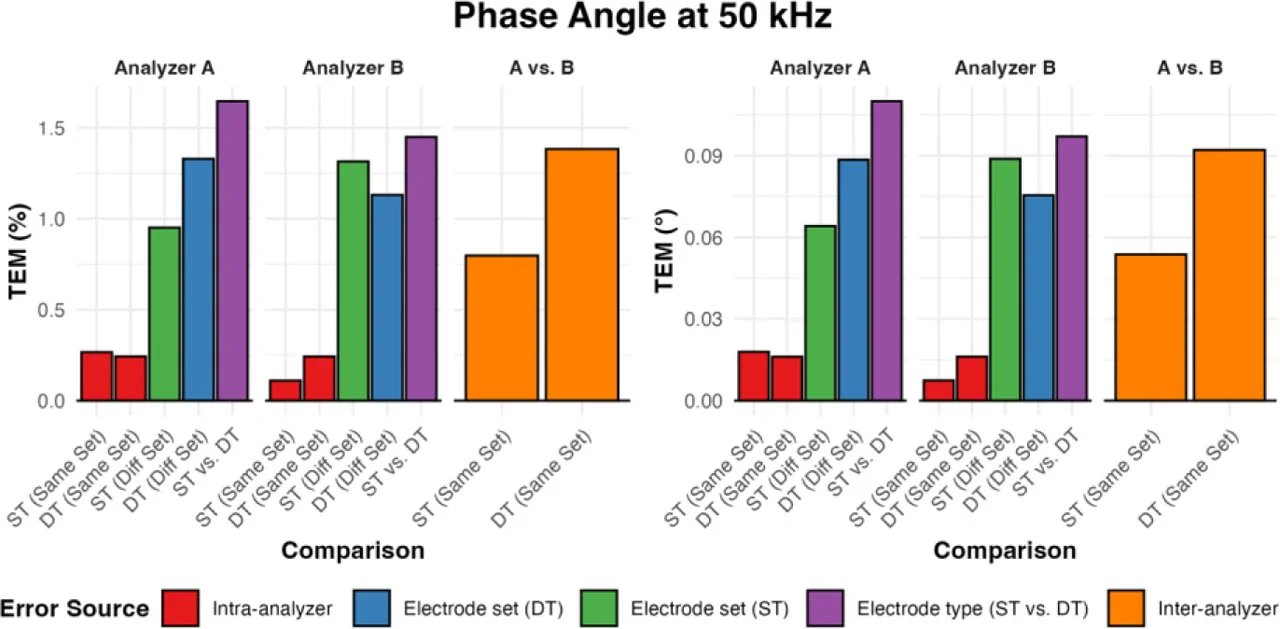
The technical error of the measurement (TEM) for phase angle (*φ*) expressed in absolute (°) and relative (%) terms for each assessment, intra-analyzer in red (1 v. 2, 3 v. 4, 7 v. 8, 9 v. 10), different set of double-tab (DT) electrodes in blue (7 v. 11 and 9 v. 12), different set of single-tab (ST) electrodes in green (1 v. 5 and 3 v. 6), comparison of the different electrode types single vs double-tab electrodes in purple (1 v. 7 and 3 v. 9), and inter-analyzer in orange (7 v. 9 and 1 v. 3).

Results for *φ* were similar to *R_50_*, with comparisons of different electrode types displaying larger TEMs (1 v. 7 absolute TEM = 0.11°, relative TEM = 1.64%; 3 v. 9 absolute TEM = 0.10°, relative TEM = 1.45%; Figure 5, purple bars). The absolute TEM for single-tab electrodes ranged from 0.01 – 0.09°, with relative TEM values of 0.11 – 0.95% (Figure 5, red (ST), green, and orange (ST) bars). For double-tab electrodes, the relative TEM values were 0.24 – 1.38%, with absolute TEM ranging between 0.02 – 0.09° (Table 2; Figure 5, red (DT), blue, and orange (DT) bars).

### Total Body Water (TBW)

**Figure 6.**
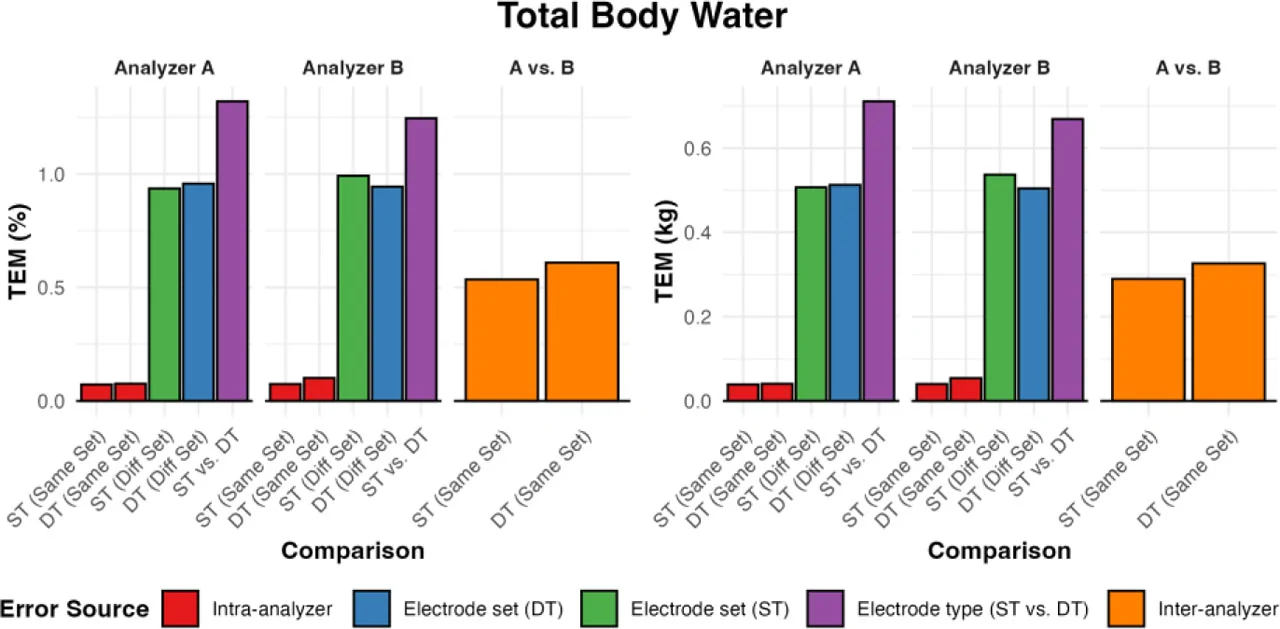
The technical error of the measurement (TEM) for total boy water (TBW) expressed in absolute (kg) and relative (%) terms for each assessment, intra-analyzer in red (1 v. 2, 3 v. 4, 7 v. 8, 9 v. 10), different set of double-tab (DT) electrodes in blue (7 v. 11 and 9 v. 12), different set of single-tab (ST) electrodes in green (1 v. 5 and 3 v. 6), comparison of the different electrode types single vs double-tab electrodes in purple (1 v. 7 and 3 v. 9), and inter-analyzer in orange (7 v. 9 and 1 v. 3).

The ranges of absolute TEM values for TBW were 0.03 – 0.38 kg for both single- and double-tab electrodes (Table 3; Figure 6). The single-tab relative TEM values were between 0.07 – 0.98% (Figure 6, red (ST), green, and orange (ST) bars) while double-tab ranged from 0.07 – 0.99% (Figure 6, red (DT), blue, and orange (DT) bars). Across all assessments, the immediate test-retest assessments (i.e., intra-analyzer error; Figure 6, red bars) displayed the smallest amount of error (absolute TEM = 0.03 – 0.04 kg, relative TEM = 0.07 – 0.10%), while the comparisons of different electrode types utilizing the same analyzer (i.e., 1 v. 7 and 3 v. 9) demonstrated larger errors (absolute TEM = 0.51 – 0.53 kg, relative TEM = 1.32 – 1.37%; Figure 6, purple bars).

**Table 3.** The absolute and relative technical error of the measurement for each of the body composition variables – total body water (TBW), fat-free mass (FFM), and fat mass (FM). See Figure 2 for assessment definitions.

**Assessment Comparison**	**Total Body Water (TBW)**		**Fat Free Mass (FFM)**		**Fat Mass (FM)**	
	Sample Mean ± SD 53.9 ± 6.3 kg		Sample Mean ± SD 73.6 ± 8.6 kg		Sample Mean ± SD 26.4 ± 8.6 kg	
	**Abs. TEM (kg)**	**Rel. TEM (%)**	**Abs. TEM (kg)**	**Rel. TEM (%)**	**Abs. TEM (kg)**	**Rel. TEM (%)**
1 v 2	0.03	0.07	0.05	0.07	0.05	0.21
1 v 3	0.19	0.50	0.40	0.54	0.40	1.52
3 v 4	0.03	0.08	0.05	0.07	0.05	0.21
1 v 5	0.38	0.96	0.69	0.94	0.69	2.66
3 v 6	0.38	0.98	0.73	0.99	0.73	2.81
7 v 8	0.03	0.07	0.06	0.08	0.06	0.21
7 v 9	0.21	0.54	0.45	0.61	0.45	1.66
9 v 10	0.04	0.10	0.07	0.10	0.07	0.27
7 v 11	0.37	0.96	0.70	0.96	0.70	2.61
9 v 12	0.38	0.99	0.69	0.94	0.69	2.55
1 v 7	0.53	1.37	0.97	1.32	0.97	3.68
3 v 9	0.51	1.32	0.91	1.24	0.91	3.44

### Fat-Free Mass (FFM)

**Figure 7.**
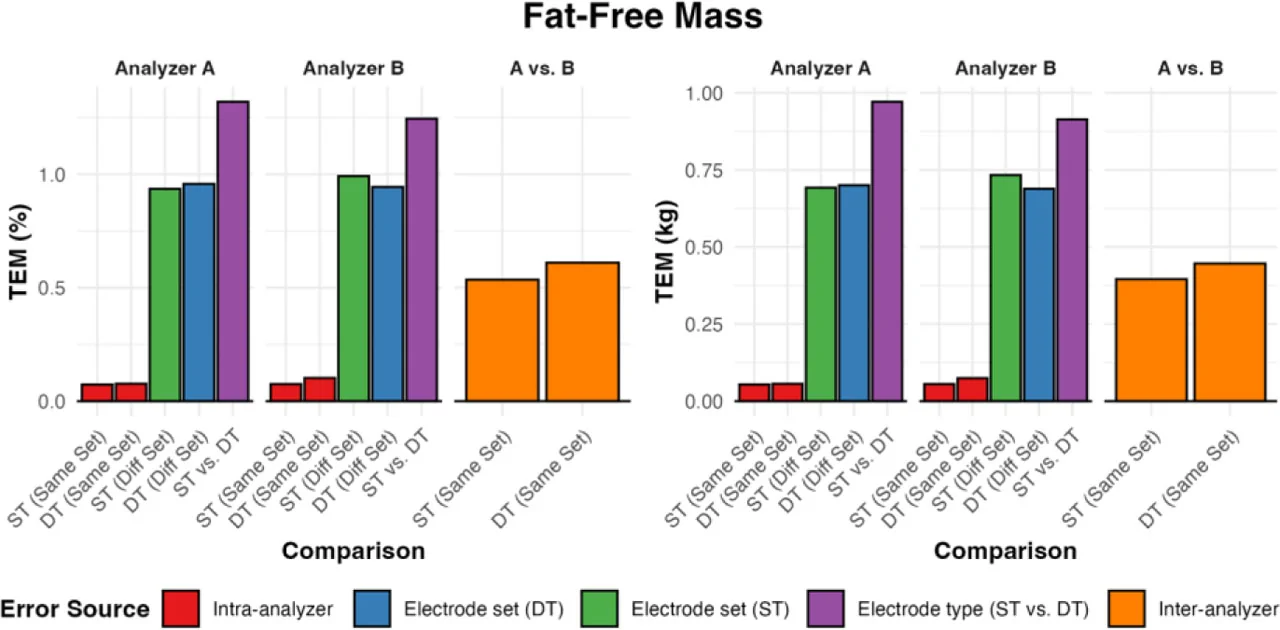
The technical error of the measurement (TEM) for fat-free mass (FFM) expressed in absolute (kg) and relative (%) terms for each assessment, intra-analyzer in red (1 v. 2, 3 v. 4, 7 v. 8, 9 v. 10), different set of double-tab (DT) electrodes in blue (7 v. 11 and 9 v. 12), different set of single-tab (ST) electrodes in green (1 v. 5 and 3 v. 6), comparison of the different electrode types single vs double-tab electrodes in purple (1 v. 7 and 3 v. 9), and inter-analyzer in orange (7 v. 9 and 1 v. 3).

FFM assessments displayed similar results as TBW, where the intra-analyzer assessments (Figure 7, red bars) displayed small absolute TEMs around 0.06 kg (relative TEMs ≤0.10%) while comparisons of different electrode types displayed larger TEMs around 0.95 kg (absolute) and 1.3% (relative) (Figure 7, purple bars). The absolute TEM for FFM for the single-tab assessments ranged from 0.05 – 0.73 kg, with relative TEM values of 0.07 – 0.99% (Figure 7, red (ST), green, and orange (ST) bars). For the double-tab assessments, corresponding TEM values were 0.06 – 0.70 kg and 0.08 – 0.96%, respectively (Table 3; Figure 7, red (DT), blue, and orange (DT) bars).

### Fat Mass (FM)

**Figure 8.**
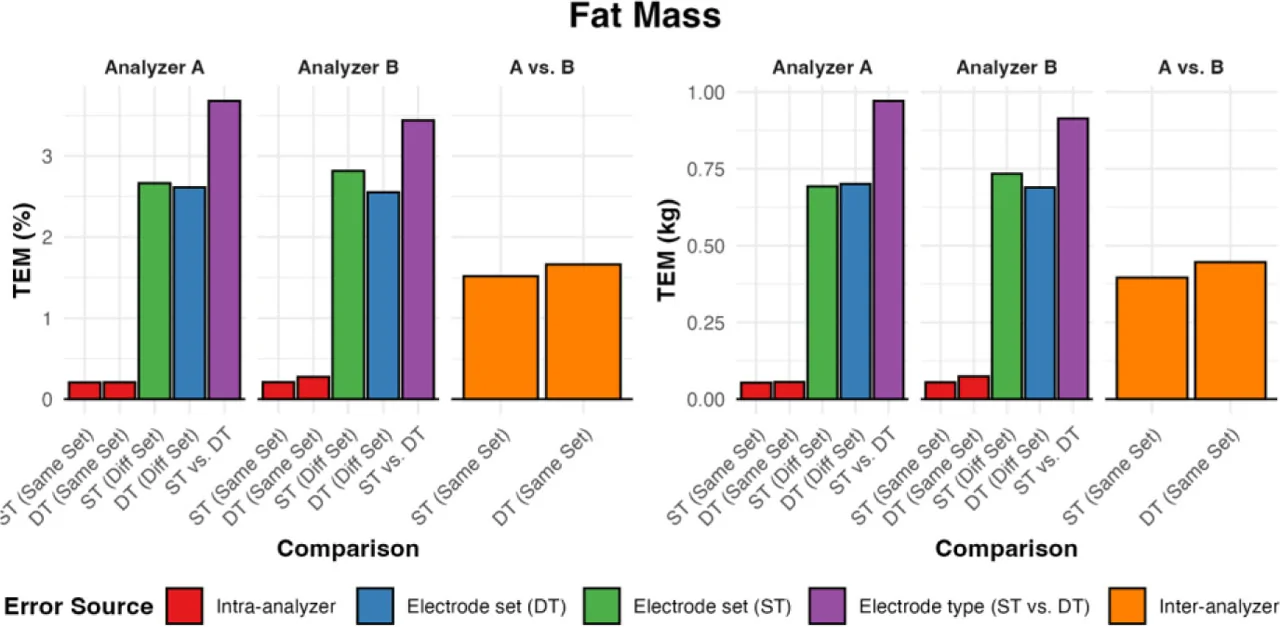
The technical error of the measurement (TEM) for fat mass (FM) expressed in absolute (kg) and relative (%) terms for each assessment, intra-analyzer in red (1 v. 2, 3 v. 4, 7 v. 8, 9 v. 10), different set of double-tab (DT) electrodes in blue (7 v. 11 and 9 v. 12), different set of single-tab (ST) electrodes in green (1 v. 5 and 3 v. 6), comparison of the different electrode types single vs double-tab electrodes in purple (1 v. 7 and 3 v. 9), and inter-analyzer in orange (7 v. 9 and 1 v. 3).

For single-tab electrodes, the absolute TEM for FM ranged from 0.05 – 0.73 kg, and relative TEM values were 0.21 – 2.81 % (Table 3, Figure 8, red (ST), green, and orange (ST) bars). Double-tab electrodes demonstrated absolute TEM values of 0.06 – 0.70 kg, with relative TEM values ranging from 0.21 – 2.61% (Figure 8, red (DT), blue, and orange (DT) bars). Similar to the results from the other body composition variables, the largest amount of error was observed when comparing single- versus double-tab electrodes using the same analyzer, which produced relative TEMs of 3.44% to 3.68% (Figure 8, purple bars). The smallest relative TEMs were observed for the immediate test-retest assessments (relative TEM = 0.21 – 0.27%; Figure 8, red bars).

## Discussion

The purpose of this study was to establish the technical error of measurement attributable to individual components of BIS assessments when evaluating raw bioimpedance, body fluids, and body composition. Using two different BIS analyzers from the same manufacturer, multiple sets of single-tab and double-tab electrodes were used in evaluations of resistance (R_50_), reactance (Xc_50_), phase angle (*φ*), total body water (TBW), fat-free mass (FFM), and fat mass (FM). This work extends a prior investigation (5), which examined the reproducibility and validity of BIS when employing different electrode configurations (i.e., single- and double-tab) to assess resistance and TBW. Our study examined both inter- and intra-analyzer error across multiple sets of single- and double-tab electrodes to further isolate sources of technical error. In the current investigation, across all variables (i.e., R_50_, Xc_50_, *φ*, TBW, FM, FFM), the lowest amount of error was exhibited in the intra-analyzer assessments (i.e., immediate test-retest) for both analyzers (i.e., A and B) and electrode types (single- and double-tab) (Figure 3 – 5; Table 2). Moreover, for the raw bioelectrical variables (i.e., R_50_, Xc_50_, *φ*), the highest amount of error for R_50_ and *φ* were displayed when assessments changed from single-tab to double-tab electrodes with the same analyzer. Specifically, for R_50_ in assessment comparison 3 v. 9, which utilized device B and compared the single-tab configuration to the double-tab configuration, the absolute TEM was 7.92 Ω (relative TEM = 1.42%). Similarly, for *φ* in assessment comparison 1 v. 7, the largest amount of error (absolute TEM = 0.11°; relative TEM = 1.64%) was noted when comparing across electrode types for device A. Interestingly, when assessing Xc_50_, the inter-analyzer error (i.e., changing from analyzer A to B) displayed the largest amount of technical error specifically for the double-tab electrodes (i.e., assessment 7 v. 9, absolute TEM = 1.07 Ω, relative TEM = 1.68%). This indicates differential effects of technical error sources on individual components of raw bioimpedance. Conversely, for measures of body composition (TBW, FM, FFM), the largest amount of error for all variables was observed for the assessments that employed the two different electrode types while utilizing the same analyzer (i.e., assessments 1 v. 7 and 3 v. 9; Figure 6 – 8; Table 3). However, as expected, the comparisons yielding the lowest errors were the same for all raw bioelectrical, body fluid, and body composition variables (i.e., intra-analyzer; immediate test-retest). The differences in measurements using single-tab and double-tab electrodes could indicate that these electrodes have different intrinsic impedance even though both styles have similar gel contact areas and are expected to have similar types of gel (but could have differences because of electrode age or manufacturing). Different intrinsic impedance has been previously reported by Nescolarde et al. to effect bioimpedance vectors, which is a method for representing both resistance and reactance for analysis (15). Different intrinsic impedances may influence how a bioimpedance instrument injects current and measures voltage of a connected tissue but requires knowledge of the underlying instrument circuitry to evaluate (which is beyond the scope of this work). Further studies should measure the intrinsic impedance of electrodes. Collectively, these results help isolate the primary sources of technical error when using BIS and indicate expected magnitudes of errors in a controlled laboratory setting.

R and Xc are the two primary components of bioelectrical impedance. While Xc is an indicator of cell membrane integrity and is related to the delay of the electrical flow by the cell membrane and tissue interfaces, R is determined by opposition of electrical flow from the intracellular and extracellular water, therefore reflecting cellular hydration (11, 16). Variable Xc and R values are expected when assessing different populations due to the unique physical characteristics influencing the capacitive elements of cell membranes, ionic substances, and properties of intracellular and extracellular fluid (17). However, when shifting focus from population level comparisons to methodological reliability, the consistency of Xc and R across repeated measures using varied technical configurations becomes the primary concern, as variability in analyzer and electrode type may introduce error independent of true physiological differences. In the current investigation, the lowest amount of error was observed for Xc_50_ and R_50_ when the specific analyzer and set of electrodes remained constant (e.g., TEMs of 0.08 Ω [0.12%) and 0.26 Ω [0.05%), respectively]. These magnitudes indicate minimal technical error associated with independent assessments when all testing components are held constant. In contrast, the highest technical errors for Xc_50_ were observed when different analyzers were used, despite retaining the same set of double-tab electrodes (absolute TEM = 1.07 Ω; relative TEM = 1.68%), indicating that the analyzer itself is a non-negligible source of error for Xc assessments, even when analyzers are the same model from a single manufacturer. For R_50_, the largest error was observed when comparing single- to double-tab electrodes (absolute TEM = 7.92 Ω; relative TEM = 1.42%), with the magnitude of error being potentially meaningful. The divergence between R and Xc for the largest sources of technical error was a notable finding. While Xc_50_ measurement error was more sensitive to the changes in devices (i.e., inter-analyzer) for the double-tab electrode assessment, R_50_ was more susceptible to error based on the electrode type employed. This pattern is consistent with the different physical basis of the respective parameters. R_50_ is reflective of the ohmic resistance of the fluid pathway between electrodes. Changes in the electrode type or the contact area may proportionally affect the measured resistance. This is consistent with a previous study (18), where contact resistance between the electrode and the skin produced relative changes in the impedance values. Moreover, Xc_50_ represents a comparatively small signal relative to R_50_ (Figure 1) and therefore may be more susceptible to inter-analyzer differences in signal processing than the electrode themselves (19, 20). The dissociation between Xc_50_ and R_50_ sensitivity sources may have implications for measurement standardization, although it is generally ideal for all components of assessments (e.g., specific analyzer, electrode type, etc.) to be standardized in order to minimize technical errors.

A small number of previous studies have examined the influence of BIS electrode configuration on the reliability or consistency of assessments (5, 13). Using the same BIS analyzer employed in the present study (ImpediMed SFB7), Moon et al. (5) compared technical errors from ‘single-site’ (single-tab) electrodes and ‘fixed distance’ (double-tab) electrodes. However, the specific anatomical sites used varied by electrode types. While the double-tab electrodes were spaced 5 cm apart and physically connected, as in the present study, the single-tab electrodes were placed at ‘standard sites’ (hand electrodes: (1) dorsal surface of the metacarpal phalangeal joint and (2) 1 cm proximal to the knuckle of the middle finger; foot electrodes: (1) dorsal surface of the metatarsal phalangeal joint and (2) 1 cm proximal to the joint of the second toe).

As such, both the electrode type and anatomical location varied in the study of Moon et al., making it impossible to fully isolate the effect of electrode type independent of the anatomical location of the electrodes. Nonetheless, it was observed that standard error of the measurement (SEM) values for R_zero_ (resistance as the current frequency approaches zero) and R_inf_ (resistance as current frequency approaches infinity) were slightly higher with the double-tab fixed-distance placement as compared to the single-tab configuration (R_zero_: 16.79 vs. 15.35 Ω; R_inf_: 9.85 vs. 9.25 Ω). However, when calculating R_i_ (intracellular resistance), the SEM was lower for the double-tab electrodes as compared to the single-tab electrodes (50.71 vs. 58.27 Ω). When the raw bioimpedance values were used to estimate TBW within the manufacturer’s software, Moon et al. found that the SEM was reduced by use of fixed-distance double-tab electrodes in males (0.81 L vs. 1.13 L with single-tab) but not females (0.47 L for both electrode types), although females displayed lower SEM values overall. The findings in our current investigation and Moon et al. (5) are consistent in demonstrating that small variations in electrode configuration may have a meaningful impact in resistance values and downstream estimates of body fluids or body composition.

Phase angle (*φ*) is a physiological index of cell mass and cell membrane integrity, which is dependent on age, sex, and fluid distribution. Higher *φ* values are associated with greater cellular membrane integrity and function (11). To facilitate evaluation of *φ*, reference values have been established in a large sample of ~2000 individuals, where the average *φ* value was 6.93 ± 1.15° (12). Although there is not a universal accepted threshold for *φ* to define a meaningful practical change, changes should be interpreted in relation to the expected variability of the measure and the magnitude of the technical error. In a meta-analysis conducted by Mattiello, Amaral (21), the authors noted that mean *φ* for adults was ~6°, but individual *φ* may decrease due to poorer cellular integrity and declines in soft tissue mass (12, 22). In the current investigation, the average *φ* was 6.70 ± 1.01°, and TEM values for *φ* ranged from 0.01 to 0.11° (0.11 to 1.64%). Notably, the two comparisons with the largest amount of error for *φ* (i.e., comparisons 1 v. 7 and 3 v. 9) compared single-versus double-tab electrodes, indicating that electrode type was the highest source of technical error for *φ*. In contrast, assessments utilizing immediate test-retest (i.e., intra-analyzer error; assessments 1 v. 2, 3 v. 4, 7 v. 8, 9 v. 10) displayed very small errors (absolute TEM = 0.01 – 0.02°; relative TEM = 0.11 – 0.27%) for both analyzers. Although our observed TEM values are low compared to average *φ* values (i.e., 6.93 ± 1.15°), the upper end of TEM values (~1.6%; ~0.11°) is large enough to potentially obscure the detection of small changes in *φ* due to physiological processes or lifestyle interventions. As such, technical standardization, particularly of electrode type, is necessary to improve the likelihood of detecting small but real changes. This is a critical methodological consideration for future investigations, particularly longitudinal studies tracking *φ* over time, where technical error may contribute to data ‘noise’ or be misinterpreted as true physiological changes.

Total body water (TBW) is the sum of the masses of intracellular and extracellular water. TBW varies depending on an individual’s age, sex, body size, and body composition (23). Based on the relationship between bioimpedance and body fluids, raw bioimpedance is often used to predict TBW, which may be subsequently used to estimate body composition. The present study allowed for a direct examination of how differences in electrode type and analyzer influence the reliability of BIS TBW estimates. When assessing TBW, the largest amount of error was exhibited in assessments 1 v. 7 (absolute TEM = 0.53 kg) and 3 v. 9 (absolute TEM = 0.51 kg), which compared the error of the different electrode types (i.e., single- vs double-tab) within each analyzer. Moreover, as observed with other outcomes, all assessments that examined the immediate test-retest reliability assessing the intra-analyzer technical error displayed the smallest amount of technical error for TBW. Therefore, in agreement with the results from the raw bioelectrical impedance variables, the main source of error is observed when comparing the reliability between the single-tab to the double-tab electrodes. These TBW results build upon validity and electrode placement considerations previously established for this BIS analyzer (5, 6).

As TBW serves as a foundational component in the estimation of body composition, its reliability has direct implication on both FFM and FM. In healthy individuals, water constitutes approximately 73% of FFM (24), and FM can be calculated as the difference between total body mass and FFM. When using this body composition estimation paradigm, any errors observed in TBW estimation are propagated to body composition outcomes like FFM and FM. Corroborating this relationship, the present investigation observed similar results between FFM and FM. Moreover, TBW is often used in FFM estimation based on the assumed hydration constant of 0.73 (TBW/FFM), with FM subsequently derived from BM and FFM. Accordingly, small differences in the raw electrical measurements can become increasingly consequential as they propagate through the estimation process for body composition variables (25). This, along with differences in absolute size of body compartments, may help explain why electrode configuration produced a greater relative effect on FM. For TBW, intra-analyzer error (i.e., immediate test-retest) exhibited the lowest amount of error (FFM and FM absolute TEM = 0.05 – 0.07 kg; FFM relative TEM = 0.07 – 0.1%; FM relative TEM = 0.21 – 0.27%), while the largest amount of error was observed for the assessments that employed the two different electrode types (average relative TEMs of 1.28% and 3.56%, respectively, for FFM and FM). The magnitude of these errors may be acceptable for simple cross-sectional classifications but may be sufficient to confound longitudinal evaluations, particularly when small changes are being evaluated.

Although meticulous methods were employed in the present investigation, this study is not without limitations. First, there is the potential for simple human error when placing the single-tab electrodes. While all single-tab electrodes were placed at a fixed distance of 5 cm, which was measured for each assessment, there could still be potential error involved when placing the electrodes. Second, our results are specific to the ImpediMed SFB7 analyzer, which may limit the generalizability to other devices. In addition, our study sample consisted of generally healthy adults, indicating that our results may not be reflective of populations with disease, altered hydration status, or groups who have body composition characteristics which differ from our current sample. Further research is needed to determine whether similar findings are observed in these populations. Lastly, bioimpedance assessments were performed in a fixed non-randomized sequence rather than a randomized order. Although all participants underwent identical pre-assessment standardization and testing procedures were completed within approximately 15 minutes, a minor time dependent effect associated within sustained supine positioning cannot be excluded. Previous work conducted by Ducharme, et al. (26) suggested that supine rest between 15 – 30 minutes may influence bioimpedance derived measures through fluid redistribution, although these finding were obtained using a different bioimpedance technology than the one employed in our current investigation. Future investigations should evaluate the effects of time-dependent supine positioning utilizing the SFB7 as well as randomization of assessment order to reduce potential influences from the order of assessments. Despite these considerations, to our knowledge, this study was the first to assess the technical error of measurement between single- and double-tab electrodes while utilizing two different SFB7 analyzers with multiple assessments in a healthy population.

Overall, when assessing raw bioelectrical variables, body fluids, and body composition utilizing bioimpedance technology, it is recommended that an identical setup is used for all tests, including the same specific device and electrode type. Although most technical errors were relatively small in the present investigation, the comprehensive evaluation of technical components of bioimpedance assessments allows for a better understanding of the sources of error specific to this technology. Additionally, the magnitude of some technical errors is sufficient to confound the detection of small longitudinal changes in outcomes. Therefore, it is important to maintain consistency and standardization throughout all bioimpedance assessments. Future investigations should continue to assess the technical error of bioimpedance in different environments outside of a controlled laboratory, as well as evaluate different analyzers to determine the extent to which performance varies across manufacturers and models.
